# Plasticity in the Neonatal Brain following Hypoxic-Ischaemic Injury

**DOI:** 10.1155/2016/4901014

**Published:** 2016-03-07

**Authors:** Eridan Rocha-Ferreira, Mariya Hristova

**Affiliations:** UCL Institute for Women's Health, Maternal & Fetal Medicine, Perinatal Brain Repair Group, London WC1E 6HX, UK

## Abstract

Hypoxic-ischaemic damage to the developing brain is a leading cause of child death, with high mortality and morbidity, including cerebral palsy, epilepsy, and cognitive disabilities. The developmental stage of the brain and the severity of the insult influence the selective regional vulnerability and the subsequent clinical manifestations. The increased susceptibility to hypoxia-ischaemia (HI) of periventricular white matter in preterm infants predisposes the immature brain to motor, cognitive, and sensory deficits, with cognitive impairment associated with earlier gestational age. In term infants HI causes selective damage to sensorimotor cortex, basal ganglia, thalamus, and brain stem. Even though the immature brain is more malleable to external stimuli compared to the adult one, a hypoxic-ischaemic event to the neonate interrupts the shaping of central motor pathways and can affect normal developmental plasticity through altering neurotransmission, changes in cellular signalling, neural connectivity and function, wrong targeted innervation, and interruption of developmental apoptosis. Models of neonatal HI demonstrate three morphologically different types of cell death, that is, apoptosis, necrosis, and autophagy, which crosstalk and can exist as a continuum in the same cell. In the present review we discuss the mechanisms of HI injury to the immature brain and the way they affect plasticity.

## 1. Introduction

Oxygen deprivation before or around the time of birth often results in hypoxia-ischaemia-induced brain damage, which remains a common cause of neonatal brain injury and affects 1 to 3 per 1000 live births in developed countries with incidence increased up to 26 per 1000 live births in the developing world [1]. The pattern of injury depends on the level of the development of the brain and on the severity of the insult, which both affect the selective regional vulnerability, as well as the subsequent clinical manifestations. In preterm infants (<32 weeks of gestation) periventricular white matter is particularly vulnerable to hypoxia-ischaemia (HI) resulting in a selective pattern of injury characterised with motor, cognitive, and sensory deficits with cognitive impairment significantly associated with early gestational age and cortical visual impairment particularly common in infants with severe preterm insult. However, in term infants severe HI causes selective damage to the sensorimotor cortex, basal ganglia, thalamus, and brain stem.

Despite the advances in neonatal healthcare, the increased understating of the pathophysiology of hypoxic-ischaemic brain injury, and the introduction of therapeutic hypothermia as standard care for moderate to severe birth asphyxia, HI continues to lead to significant long-term neurodisabilities or mortality. Birth asphyxia causes an annual estimate of one million or 23% of all neonatal deaths worldwide [2, 3].

The aim of this review is to summarize the current knowledge on the pathogenesis of neonatal HI brain damage, including the excito-oxidative cascade, the selective regional and cellular vulnerability, mitochondrial damage, cell death continuum and crosstalk following HI, and its effects on the subsequent brain development and plasticity.

## 2. Pathogenesis of Hypoxia-Ischaemia

**Figure 1:**
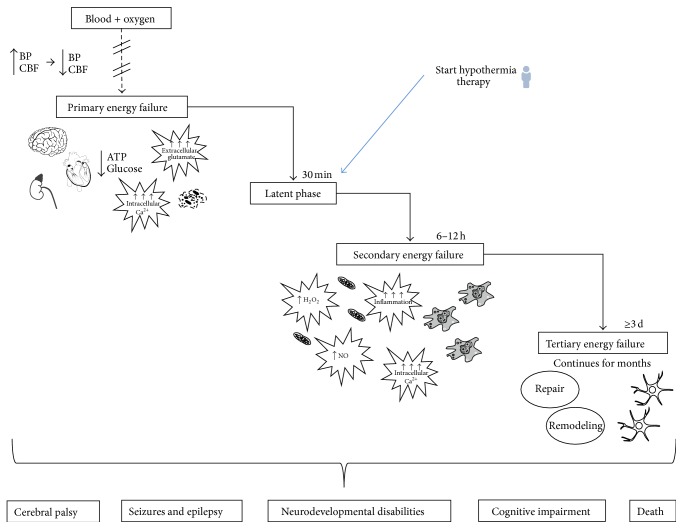
Schematic overview of hypoxia-ischaemia pathology. Disruption of blood and oxygen supply results in an initial increase in blood pressure and cerebral blood flow with redistribution favoring the brain, heart, and adrenal glands, as well as reduction in ATP due to limited glucose availability. This results in intracellular accumulation of calcium and cell membrane depolarisation and initial mostly necrotic cell death. During the latent/recovery phase there is normalization of homeostasis. However, if the initial insult is prolonged or severe, this may result within hours in a secondary delayed energy failure, due to disruption of mitochondria function as a result of excitotoxicity, inflammation, and continual uptake of intracellular calcium as well as release of oxygen reactive species. It is during the secondary energy failure that most cell death occurs, with predominant apoptosis. A tertiary phase may occur within days after initial injury and continues for months. This involves late cell death, astrogliosis, remodelling, and repair. Hypothermia, the only clinical treatment available for neonatal encephalopathy, targets the latent phase.

It is now well established that HI brain injury is a syndrome that evolves over days, even weeks [4]. During normal conditions, the human brain has a high requirement for oxygen and glucose, normally used in oxidative phosphorylation to produce adenosine triphosphate (ATP). During a HI episode, oxidative phosphorylation is rapidly reduced leading to primary energy failure in glutamatergic neurons. The subsequent brain injury will depend on the severity and duration of the HI insult, where with the assistance of magnetic resonance imaging (MRI) the two main patterns of brain injury have been described: basal ganglia thalamus (BGT) and watershed. BGT has mostly been associated with infants suffering an acute profound HI episode, whereas watershed predominant pattern is more frequently seen in infants with partial prolonged HI. However, studies have also shown a mixture of these two patterns occurring, as well as the presence of other patterns of brain injury [5]. During a HI insult, the fetus is able to maintain a temporary degree of homeostasis by reduction of nonobligatory energy consumption favoring the heart, brain, and adrenal glands, as well as suppression of neuronal activity and short period maintenance of anaerobic respiration [6–9]. However, this reduced ATP availability/production results in rapid consumption of glucose reserves, followed by severe metabolic acidosis as a consequence of lactic acid accumulation [10–13]. This is followed by successful resuscitation and normalization of high-energy-containing phosphate compounds, such as phosphocreatine and nucleotide triphosphates. However, in HI brain injury this recovery phase is short lived and a second wave of secondary energy failure starts from as early as 6 hours after initial injury [5]. A schematic overview of hypoxia-ischaemia pathology is presented in Figure 1.

## 3. Excito-Oxidative Cascade

One of the earliest events occurring during the evolution of HI injury is the excito-oxidative cascade. As a result of reduction in high-energy phosphate metabolism, subsequent increase in cerebral lactic acidosis leads to cell membrane ionic transport failure. As the Na^+^/K^+^ pumps stop functioning, accumulation of Na^+^, Ca^2+^, and Cl^−^ within the cell occurs. This calcium overload causes activation of lipases, proteases, and endonucleases leading to destruction of the cellular skeleton [14]. Rat models of neonatal HI have shown that this cytoplasmic accumulation of calcium and severe cell swelling results in necrosis and eventual activation of multiple cascading events leading to further cell death at a later stage [15–17]. Additionally, this change in neuronal membrane voltage results in depolarisation and excessive presynaptic release of glutamate, which is normally removed by perisynaptic glia glutamate reuptake pumps during aerobic metabolism of glucose [18]. As ischaemia reduces glucose availability, reuptake of glutamate is severely depleted causing overactivation of the glutamate receptors. This excitotoxicity is present in multiple highly metabolic brain regions, including the perirolandic cerebral cortex, thalamus, and putamen, as well as in the cerebrospinal fluid [19–21]. Neurons and oligodendrocyte progenitors are among the cells that express glutamate receptors, including the ionotropic ligand-gated ion channels N-methyl-D-aspartate (NMDA) receptor, a transmembrane receptor that allows entry of calcium and sodium into the cell and potassium out; *α*-amino-3-hydroxy-5-methyl-4-isoxazole (AMPA) receptor, which is both a glutamate receptor and a cation channel for sodium and potassium; and kainate receptors, also permeable to sodium and potassium [22–25]. Additionally, glutamate also activates metabotropic receptors, known as regulators of intracellular G-protein signal cascades [14]. Overstimulation of glutamate ionotropic receptors and subsequent substantial increase of calcium influx into neurons result in mitochondria dysfunction [21, 26]. Blockage of NMDA and AMPA receptors has been reported to significantly reduce brain injury in a rat model of HI injury [27].

## 4. Inflammation

HI brain injury induces an immediate inflammatory response, which has been shown to last for days and even weeks following initial insult [28, 29]. The initial inflammatory response's purpose is to target the damaged region and involves recruitment and activation of immune cells and active removal of damaged cells/debris and lipids, in an attempt to reduce infection. This is followed by a switch from pro- to anti-inflammatory immune profile to stimulate healing and tissue repair.

Initial immune response is characterised by activation of microglia, the innate immune cells of the brain, and migration of peripheral macrophages, monocytes, and neutrophils into the site of injury. Microglia cells also contribute to the cytotoxic damage following HI. It is thought that stressed neurons activate microglia as early as 2 hours following injury, which then produce and release proinflammatory cytokines such as IL-1*β* and TNF*α* [30–32], proteases, complement factors, activation of respiratory burst, and NMDA-mediated toxicity, thus contributing to secondary energy failure damage [33, 34]. Additionally, microglial activation and subsequent release of cytokines has been directly linked with axonal injury, that is, white matter damage [35–37]. Astrocytes, which constitute the majority of glia cells in the brain, play an important role in glutamate uptake and metabolism, constitute part of the blood-brain barrier, and form the glial scar surrounding the lesion site following injury. The latter helps reduce injury but also delays functional recovery [38–41]. Furthermore, reactive astrocytes also release proinflammatory cytokines such as IL-6 and TNF*α* [42–44]. Neutrophils have been shown to accumulate in ischaemic brain tissue of neonatal mice in a much smaller extent than in adults, appearing only 42 hours after injury, and mostly present within blood vessels with limited and transient brain infiltration following HI [45, 46]. Interestingly, neutrophil-target neuroprotection only demonstrated beneficial effects when neutropenia was induced prior to HI and not after [47]. Adaptive immune cells, such as lymphocytes, have reduced infiltration in the initial stage of brain inflammation [46, 48], with a study using middle cerebral artery occlusion demonstrating T cell infiltration 24 hours after injury, which persisted up to 96 hours [49]; this reduced response is thought to be partially a result of lymphoid progenitor cells immaturity, as blood mononuclear cells remain largely undifferentiated during the early neonatal period with diminished expression of surface markers [50]. Nonetheless, in the chronic long-term inflammatory response to HI, infiltration of CD4 T cells was shown to occur 7 days after injury, persisting for up to 35 days after HI [46].

Inflammatory cytokines are highly associated with HI injury and are released by both innate and brain infiltrating cells [51]. IL-1*β*, IL-6, and TNF*α* release lead to further synthesis of other cytokines and adhesion molecules, promoting infiltration of leukocytes, increasing recruitment of immune cells into the site of injury, and exacerbating damage [46, 47, 52, 53]. Cytokines are also inducers of mediators of cellular damage such as ROS, as well as cell death: FasL, TNF, TRAIL, and TWEAK [54].

## 5. Selective Regional and Cellular Vulnerability following Neonatal Hypoxia-Ischaemia

HI does not result in a uniform or global brain injury but causes selective damage to different brain structures, which, as previously mentioned, depends on the severity and duration of the insult as well as on the developmental stage of the brain when it occurs [55–57]. The immature brain is relatively resistant to hypoxia alone compared to the adult one due to its strong protective mechanisms such as capability to increase cerebral blood flow [55]. It is only when hypoxia is combined with an ischaemic event in the neonatal brain, that it causes injury developing for several days, accompanied by increased neuronal excitement with recurrent seizures and electroencephalographic defects [55]. Evidence from the clinical practice supported by MRI suggests that neonatal HI preferentially affects systems controlling tone and movement [21, 58]. In preterm infants (<32 weeks of gestation) periventricular white matter is particularly vulnerable to HI resulting in a selective pattern of injury, called periventricular leukomalacia (PVL) [21, 59], and characterised with motor, cognitive, and sensory deficits [21]. Cognitive impairment is significantly associated with early gestational age [56], with cortical visual impairment particularly common in infants with severe PVL [56]. In term infants severe HI causes selective damage to the sensorimotor cortex, basal ganglia, and thalamus [60], as well as brain stem [55]. The selective damage to cortex and basal ganglia commonly results in severe motor disability, including rigidity, impairment of mostly the upper limbs, and speech difficulties [55, 61, 62]. The pattern of injury, as previously mentioned, depends on the level of the development of the brain and on the severity of the insult, which both affect the selective regional vulnerability, as well as the clinical manifestations referred to as spastic diplegia in the case of PVL and extrapyramidal or dyskinetic cerebral palsy in near-total asphyxia [21].

Unilateral carotid artery ligation in neonatal mice and rats, combined with exposure to moderate hypoxia, causes ipsilateral ischaemic white matter injury, reproducing many anatomical features of PVL [63]. The vulnerability of the immature white matter to HI injury has been for a long time attributed to the immaturity of its vascular supply [63]. Later studies have demonstrated the natural vulnerability of oligodendrocyte progenitors and immature oligodendroglia to excitotoxic, oxidative, and inflammatory insults as a major mechanism of susceptibility to injury [63]. Several studies have investigated the early events, as well as the long-term behavioural and imaging outcomes following HI, demonstrating motor and cognitive impairment and severe cerebral abnormalities [63]. Additionally relatively mild HI insult has also been reported to result not just in immediate but also in late progression of tissue damage [64]. However, clinical and laboratory evidence suggests that both developmentally dependent patterns (term and preterm) of neonatal HI brain injury are associated with glutamate-mediated excitotoxicity [21]. Several studies confirm increased glucose metabolism in regions vulnerable to HI. Positron emission tomography (PET) of children who suffered severe hypoxic insult with subsequent permanent neurological disabilities demonstrated increased glucose metabolism in sensorimotor cortex and basal ganglia [65]. Pu et al. observed an elevated proton MR scanning peak for glutamate/glutamine in basal ganglia and thalami of infants with moderate or severe HI injury, but not in infants with mild injury or normal ones [66]. Regional changes in neuronal glucose metabolism have been related to synaptic activity rather than to changes in the neuronal cell body, thus suggesting that areas with enhanced glucose metabolism are likely to have enhanced synaptic activity [67]. These findings have been confirmed through developmental studies of glucose metabolism, showing correlation between changes in synaptic density and glucose metabolic rate, as well as through MRI studies in rodents and humans where cerebral glucose metabolism has been correlated with glutamate neurotransmitter cycling during synaptic activation [21, 68, 69]. Thus the vulnerability of selected brain regions to HI is likely to be a consequence of excessive activity of excitatory synapses [21]. It is noteworthy that the selectively vulnerable regions (somatosensory cortex, putamen, and thalamus) have been confirmed to have high metabolic rate [21] and are interconnected by functionally active excitatory glutamatergic neurons [70]. Therefore the selective vulnerability of the different regions following neonatal HI could be a consequence of their position within excitatory circuits [71]. The vulnerability of selected neuronal populations to severe asphyxia can be explained with their proximity to developing glutamatergic circuits [21] and this hypothesis is supported by data obtained from animal experiments showing obvious increase in extracellular glutamate following removal of glutamate from the synapse thus reducing delivery of glucose and depriving the perisynaptic glial transporter from energy [21]. In humans the severity of seizures and other clinical symptoms of encephalopathy following HI correlates with increased levels of glutamate, aspartate, and glycine in cerebrospinal fluid, which could be a consequence of glutamate transporter malfunction [72]. For example, Martin et al. reported early loss of astroglial glutamate transporter in areas with selective neuronal degeneration in a piglet model of asphyxia [60, 73]. Increase of extracellular concentrations of glutamate and other excitotoxic amino acids such as glycine, combined with membrane depolarisation due to mitochondrial dysfunction, contributes to opening of the NMDA receptor channels, allowing an influx of sodium and calcium and subsequent intracellular injury [21]. Murugan et al. observed that hypoxia-induced excess levels of extracellular glutamate prevented its uptake by astroglial excitatory amino acid transporter and augmented the expression of functional astroglial NMDA receptor [74]. Thus increase in gap junction proteins between astroglia and oligodendroglia following hypoxia contributes to the spreading of NMDA receptor-mediated excitotoxic calcium signals into oligodendrocytes triggering oligodendroglial apoptosis and contributing to neonatal periventricular white matter damage [74].

NMDA channel blockers, such as dizocilpine (MK-801), magnesium (endogenous cationic NMDA channel blocker), and other NMDA-antagonist drugs, including ketamine and dextromethorphan, have proven neuroprotective in rodent models of neonatal HI if used before or shortly after injury; however delayed application appears less beneficial [21]. A HI event impairs ATP-dependent pumps, that is, Na-K ATPase, which triggers Na^+^ accumulation and K^+^ efflux thus gating voltage sensitive Ca-channels and stimulating reverse Ca-Na exchange leading to build-up of Ca^+^ [75]. This Ca^+^ overload is responsible for the inappropriate stimulation of Ca-dependent enzyme systems, leading to structural and functional axonal injury and abolished propagation of the action potential [75]. Therefore blockade of voltage gated Na-channels or AMPA receptors can provide protection for central axons and glia [75]. Use of AMPA-type glutamate antagonists alone during ischaemia has no effect, yet combining memantine (an NMDA receptor blocker) with an AMPA/kainate receptor blocker improved recovery of the action potential in myelinated axons after ischaemia suggesting NMDA receptor blockers as potentially useful therapeutic treatment for some white matter conditions [76].

Glutamate-mediated injury results in prolonged destruction of oligodendrocyte precursors after a HI event. NMDA receptors are present in the myelinating processes of oligodendrocytes, where the small intracellular space could lead to a rise in intracellular Ca^+^ and Na^+^ concentration in response to NMDA receptor activation. Simulated ischaemia triggers an inward current in oligodendrocytes partly mediated by NMDA receptors that can weakly be blocked by magnesium and that may contain NR1, NR2C, and NR3 subunits, suggesting oligodendroglial NMDA receptors of unusual subunit composition as a potential therapeutic target for preventing white matter damage in a variety of diseases [77]. Nevertheless, cerebral recovery and cellular reorganisation following neonatal HI have also been described, with long-term regeneration of oligodendrocyte progenitors and remyelination also taking place [63, 78]. Following HI, the precursor cells in the subventricular zone demonstrate multipotency* in vitro* and generate more neurons and oligodendrocytes* in vivo* [78–80] suggesting that the early postnatal subventricular zone is a potential source of different progenitor cells for repair, including oligodendrocyte progenitors [78]. Following moderate neonatal HI, myelin basic protein (MBP) is initially decreased in the ipsilateral hemisphere but recovers within a couple of weeks, while more severe injury results in a prolonged reduction of the levels of MBP [63]. This suggests generation of new oligodendrocytes, either migrating from the subventricular zone or arising from oligodendrocyte progenitors in the spared white and grey matter [80, 81].

Subplate neurons are a transient cell type located beneath the cortical plate. They form one of the first functional cortical circuits and are crucial for the normal visual cortical development and plasticity [56]. Subplate neurons incorporate into synaptic networks providing excitatory interconnections between neocortical layer IV and the thalamus. Human thalamocortical development begins at 22–25 weeks of gestation (GW), while synaptogenesis of the visual cortex takes place between 28 GW and birth [56].

In mice subplate neurons undergo apoptosis in the first postnatal week [82] and are mostly absent from the adult neocortex [56, 83]. In humans the peak of the subplate zone development coincides with the window of susceptibility to PVL, that is, 24 GW, decreases during the third trimester, and is absent after 6 months of age [56]. Thus damage to these neurons might play a role in the pathology of many neurodevelopmental disorders [84]. In a preterm model of HI McQuillen et al. observed complete neuronal cell death in the subplate zone, while cortical neurons were spared [56] and attributed this high subplate neuronal susceptibility to HI to early maturation, associated with an increase of NMDA-type and AMPA/kainate glutamate receptors [56]. The same group suggests that PVL disrupts the activity-dependent refinement of thalamocortical connections into mature ocular dominance columns [56], which form through activity-dependent competition for neurotrophins. As mentioned before, animal models of moderate HI resembling PVL have demonstrated only transient decrease in MBP expression due to proliferation of reactive late oligodendrocyte progenitors [85]. Visual testing of premature children with moderate PVL at 1 year of age revealed at least one abnormality in 71% of the infants; however 66% of those had normal optical radiation and visual cortex [56, 86]. This phenomenon could be explained with the selective vulnerability of subplate neurons to HI either on their own or in combination with oligodendroglial damage [56].

## 6. Mitochondrial Damage in Hypoxia-Ischaemia

When short in duration, primary energy failure phase is rapidly compensated during the reoxygenation by cerebroprotective mechanisms, with redistribution of blood flow and increase of brain, heart, and adrenal glands mediated cardiac output [6–9]. However, in more acute or prolonged reduction in blood gas exchange, or following successful resuscitation, a secondary wave of energy depletion occurs. This is associated with a substantial increase in exhaustion of cellular energy reserves (ATP), as well as a rise in lactate, pH fluctuation, and increase in oxidative stress [87, 88], as well as high calcium influx into the mitochondria matrix [89–91]. This is followed by epileptogenic activity, which can be supervised through EEG. Several different animal studies have demonstrated not only this biphasic evolution in injury, but also the fact that it is during the second energy failure phase that the majority of cellular death occurs [92–94]. This is likely to be a result of the presence of oxygen radicals, nitric oxide, inflammatory response, and excitatory amino acids. Whereas production and release of free radicals has been shown to occur during the primary injury, it is in fact during the reperfusion period that most of the oxidative markers are generated.

As previously mentioned, it is known that the brain has a high requirement for aerobic respiration, which signifies a higher rate of mitochondrial respiratory activity, thus potentiating the risk of free radicals release from this organelle. Additional sources of reactive oxygen species include nitric oxide synthase (NOS), several steps in the arachidonic acid metabolism, and compromised pathways involving xanthine and superoxide dismutase. Furthermore, HI-mediated decrease in intracellular pH may alter binding of metals, such as iron, thus increasing its catalytic activity in the Harber-Weiss reaction [95]. Brain lipids are highly enriched in polyunsaturated fatty acids (PUFAs); also many brain regions, such as the striatum, contain a high concentration of iron. This causes the brain to be highly susceptible to lipid peroxidation, destruction of cellular membrane, as well as DNA damage, degradation of protein structure, and tissue deterioration [96–98]. In correlation with these findings, a neonatal rat model of HI has shown that use of the xanthine oxidase inhibitor allopurinol prevented severe neuronal cell loss, a strong indicative of the significance of oxygen radicals in the development of secondary/delayed neuronal cell loss [99]. Additionally, a study by Millerot-Serrurot and colleagues has shown an immediate transient increase in iron levels within the hypoxic tissue of rats that underwent permanent focal ischaemia. Furthermore, iron chelation resulted in reduction of ischaemic-mediated damage [100].

Nitric oxide (NO) is synthesized within the brain from arginine, nicotinamide adenine dinucleotide phosphate (NADPH), and oxygen by NO synthase (NOS). This production is initiated by excessive glutamate release that causes coupling and activation of the NMDA receptor, allowing calcium to excessively enter into the brain cells, especially in regions such as the thalamus and basal ganglia. During HI, the mitochondria electron transport chain is interrupted, causing the H^+^ gradient in the inner membrane to dissipate, thus stopping ATP production and mitochondria depolarisation, leading to calcium accumulation within the inner membrane [101, 102]. Excessive intracellular calcium causes activation of NOS, which then produces NO, water, and citrulline. Oxidative stress leads to an excessive production of NO, which then combines with superoxide radicals to produce peroxynitrite [103], which is quickly decomposed to form NO^2+^, nitrogen dioxide, and hydroxyl radicals. This results in mitochondrial dysfunction and permeabilisation, accompanied by failure of oxidative phosphorylation [104, 105]. The NO-induced neuronal toxicity has been demonstrated in neonatal rodent models of HI, where both inhibition of NOS 1.5 h before insult in the rat and neuronal NOS (nNOS) deletion in mice demonstrated a highly protective effect, particularly in the hippocampal and cortical brain regions [106, 107]. Furthermore, nNOS and inducible NOS (iNOS) inhibition also improved long-term outcomes in another neonatal HI model [108]. Additionally, NO can impair mitochondria respiration by disrupting cytochrome oxidase/complex 4 function and complex 1, thus increasing mitochondrial production of superoxide and peroxynitrite ions, particularly during hypoxic insult [109, 110]. As aforementioned, HI injury leads to accumulation of lactic acid which is caused, in part, by mitochondria permeabilisation and loss of function, as shown in a MRI study by Fatemi and colleagues [17]. Accumulation of superoxide and peroxynitrite can increase trafficking of cytochrome C and apoptosis-inducing factor (AIF), both proapoptotic proteins, from the outer mitochondria membrane into the cytoplasm, triggering intrinsic pathway-mediated apoptosis. In neonates, the proapoptotic protein Bax initiates this outer mitochondrial membrane permeabilisation [111]. Subsequent experiments using both a neonatal mouse model of HI and an adult rat model of cerebral ischaemia have shown that administration of Bax-inhibiting peptides reduced brain injury [112, 113]. Once in the cytoplasm, cytochrome C binds to caspases triggering activation of caspase-3, which in turn initiates apoptotic-mediated DNA fragmentation [114, 115]. AIF, on the other hand, triggers non-caspase-mediated DNA fragmentation, which is associated with increased activity of poly-ADP-ribose polymerase 1 (PARP1) [116]. HI injury also induces autophagy. A study by Hoshino et al. has shown that autophagosomes present within the ischaemic border zone in the heart had a 5-fold increase in mitochondria, indicating potential mitophagy [117]. Mitochondrial biogenesis was also present in the brain of rats 6 hours after neonatal HI, which was also associated with increase in HSP60 and COXIV as well as citrate synthase activity in the neurons within the cortical border zone. This suggests an endogenous attempt for repair following HI injury [118].

## 7. Apoptosis-Necrosis Continuum following Neonatal HI

Based on biochemical and morphological criteria, cell death is usually classified as either apoptotic (Type I) or necrotic (Type III). While apoptotic cells represent the developmentally programmed cell death and are characterised by cytoplasmic condensation and shrinkage, plasma membrane blebbing, fully undamaged cytoplasmic membrane, and tightly packed chromatin clusters, necrotic cells have complete organelle disruption, swelling and tearing of the cell membrane, and widely scattered small chromatin clusters (Table 1) [119]. Both necrosis and apoptosis, as well as a third hybrid form, combining features of both necrosis and apoptosis have been registered as types of cell death after HI. The mode of cell death that cells will undergo after HI depends on the severity of the insult, the glutamate receptor subtype that has been stimulated, the degree of cellular calcium overload, the maturity of the affected cell type, as well as cellular energy depletion, and mitochondrial dysfunction [119]. Postmortem brain tissue from infants following neonatal HI injury, as well as neonatal animal models of such injury, suggests that apoptosis is more prominent in the immature compared to the adult brain [55], probably due to the fact that the former preserves more cells with capacity for apoptotic cell death and eliminates them during development. Zhu et al. show severalfold more pronounced nuclear translocation of apoptosis-inducing factor, cytochrome C release, and caspase-3 activation following HI in the immature compared to the adult brain, with hippocampal CA1 subfield shifting from apoptosis-related neuronal death at P5–P9 to necrosis related calpain activation at P21 and P60 [120]. Nakajima et al. report that more than 50% of the degenerating cells in several brain regions following HI in the neonatal rat are apoptotic [121], while following adult middle cerebral artery occlusion Li et al. observe a ratio of 1 : 6 to 1 : 13 apoptosis versus necrosis [122]. Interestingly, in many regions such as the cerebral cortex and basal ganglia the number of apoptotic cells remains high for more than a week following HI [121].

The levels of several biochemical markers of apoptosis have been reported to be increased following neonatal HI. Caspase-dependent pathways are activated to execute programmed cell death in numerous cell types and also play an important role in neurodegeneration following neonatal HI [120, 123, 124]. Johnston et al. demonstrated that, following HI insult in 7 d old rats, regions with apoptotic morphology also showed increased levels of caspase-3 [21]. Although pan-caspase inhibition in models of neonatal HI has proven neuroprotective [125, 126], this type of inhibition is not selective, because caspases, as well as being involved in programmed cell death after injury, are also crucial for the normal brain development. Inhibition of the executioner caspase-3, which precedes DNA fragmentation following neonatal HI, although moderately neuroprotective, is undesirable with respect to the important role of caspase-3 in physiological apoptosis and its effect on brain development [127]. Caspase-2 is an initiator caspase, which, similarly to caspase-3, increases in the immature brain following HI insult in an age-dependent manner [125, 126, 128]. Deletion of caspase-2 in the immature brain is neuroprotective, especially when combined with mild hypothermia [127].

Activation of caspases may be linked to calpain activation, which regulates cytoskeletal function [55, 129]. Northington et al. observed that Fas death receptor protein expression rapidly increased after neonatal HI, in line with cleavage of procaspase-8 and increase of Bax and cytochrome C, and accompanied with mitochondrial abnormalities in the thalamus, and preceded caspase-3 activation and apoptosis at 24 h after HI in the neonatal rat [48].

Despite all the biochemical markers of apoptosis observed in the neonatal brain following HI insult, several studies fail to demonstrate typical apoptotic neuropathology in the acute phase after HI [130, 131].

The term “apoptotic-necrotic continuum” has been introduced as a definition for cells exhibiting a hybrid type of cell death, combining both apoptotic and necrotic morphology following a neonatal excitotoxic insult [132]. Another term defining this hybrid type of cell death is “pathological apoptosis” introduced by Blomgren et al. and referring to cells exhibiting typical programmed cell death features, such as pyknosis, caspase-3 activation, and nuclear condensation, along with nonprogrammed cell death characteristics [133]. Apoptotic-necrotic continuum includes a variety of cell death morphologies (Table 1), such as incomplete nuclear and cytoplasmic packaging, disruption of mitochondrial integrity in areas with mitochondrial energy failure, and trafficking distresses, observed within one or more closely related regions in the neonatal brain following single insult combined with substrate depletion [119]. The apoptotic-necrotic continuum is well reported in the neonatal brain following HI injury [119, 121, 131, 134], although the exact mechanisms behind this hybrid type of cell death are not very clear and are suggested to be a consequence of interrupted apoptosis signalling due to mitochondrial structural and functional failure [119]. Northington et al. suggest that the predominant form of cell death following neonatal HI injury is the apoptosis-necrosis continuum characterised with partial activation of the caspase cascade, as well as transitional forms of cell degradation biochemical markers [119]. This would explain why within 24 h following HI event in the neonatal brain markers of apoptosis such as caspases 3 and 9 are abundant, but there is no ultrastructural evidence for apoptotic cell death [119]. HI injury is associated with an energy failure, occurring simultaneously with activation of apoptotic pathways. Decrease of ATP* in vitro* by 30–50% produces transitional cell death forms, including inhibition of nuclear condensation and DNA fragmentation [135] corresponding to the typical continuum cell death phenotype (Table 1).

## 8. Autophagy and Cell Death following Neonatal HI

**Figure 2:**
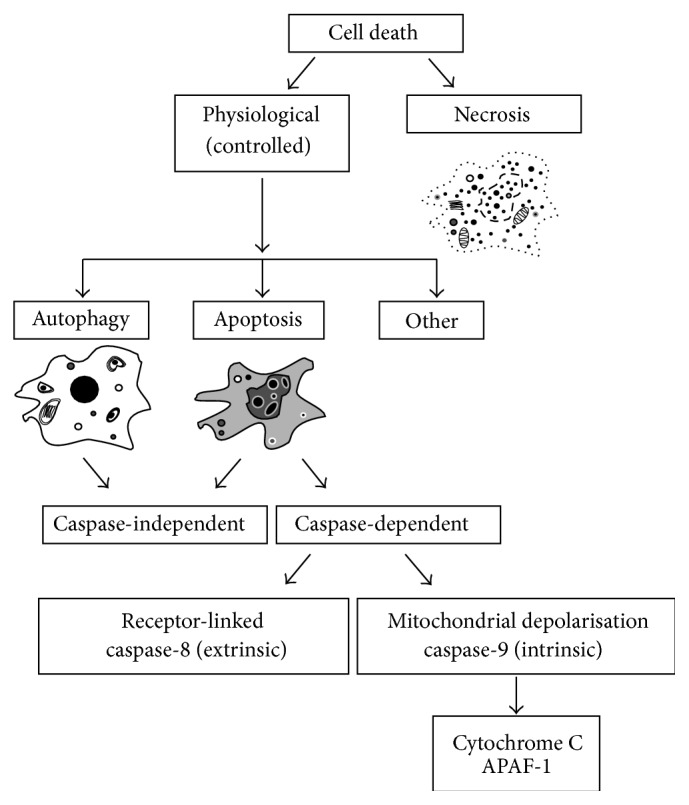
Schematic presentation of the relationship between the different types of cell death. Cell death could be controlled (physiological), including autophagy (caspase-independent) and apoptosis (caspase-dependent), or necrotic. The boundaries between apoptosis, necrosis, and autophagy are not always clear. Apoptotic death is mostly caspase-dependent; however apoptotic morphology can sometimes be registered without obvious caspase activation [136]. Caspase activation can occur through membrane receptor binding (extrinsic) or as a result of metabolic changes following mitochondrial depolarisation (intrinsic) and release of cytochrome C and APAF-1 (adapted from [136]).

Autophagy is an adaptive process through which eukaryotic cells degrade and recycle their own cytoplasm and organelles via a lysosomal system, in response to unfavourable conditions [137, 138]. Autophagy is considered to be a homeostatic nonlethal stress response protecting the cell from low nutrient supplies [138] and is classified as Type II programmed cell death [139]. A hallmark of autophagy is the formation of double-membrane autophagosomes derived from the endoplasmic reticulum, beginning with nucleation (induction) and followed by phagosome formation, subsequent autophagosome maturation, and fusion with a lysosome, succeeded by degradation or recycling of the autophagosome content [140]. There is a crosstalk and continuum between autophagic and apoptotic cell death pathways. Autophagy may proceed to apoptosis and in turn to necrosis, but autophagy can block apoptosis by sequestration of mitochondria. Extracellular or metabolic signals can trigger stress response in the cells. If the subsequent injury is repairable, the cell might undertake autophagy to sequester the damage to the organelles. However if autophagic capacity is decreased and the damage cannot be repaired or removed, autophagic cell death might occur or intrinsic apoptosis pathway might be initiated through mitochondrial polarisation and caspase-9 activation. If the injury cannot be repaired the cells might directly undergo apoptotic cell death either through intrinsic (caspase-9) or extrinsic receptor-linked (caspase-8) pathways. However caspase inhibition can alter the cell death process into autophagy [141]. A schematic summary of the relationship between the types of cell death is presented in Figure 2 [136].

Autophagy is seen in developmental and pathologic conditions and both* in vitro* and* in vivo* studies reveal that it has a significant role in the damage occurring after neonatal HI, depending on the severity of the insult, the time, and the affected region [138, 140]. For example, in a rat model of neonatal HI Ginet and collaborators demonstrated earlier induction of autophagy in cortex and CA3 hippocampus in comparison to striatum or thalamus [142]. Several studies demonstrate that dying neurons with high level of autophagy also express apoptotic features [140, 142, 143]; however that is again region specific. Following neonatal HI cell death in CA3 hippocampal neurons, for example, is associated with a more autophagic phenotype, while the CA1 hippocampal neurons have strong apoptotic characteristics [142]. Inhibition of autophagy through neuron-specific deletion of Atg7 or knockdown of Beclin-1 results in near complete protection of hippocampus in neonatal HI [144, 145], and delayed pharmacological inhibition of autophagy with 3-methyladenine in focal ischaemia proves neuroprotective in neonatal rats [138, 146]. Conversely, pretreatment with 3-methyladenine and wortmannin, both inhibitors of autophagy, reduces Beclin-1 and switches the cell death mechanism from apoptotic to necrotic; however preinsult treatment with rapamycin, resulting in enhanced autophagy, augments Beclin-1 expression, reduces necrotic cell death, and decreases brain injury [147]. Therefore neuroprotective pharmacological pretreatment despite increasing markers of autophagy can potentially provide neuroprotection [140, 147].

More* in vivo* studies, along with computational analysis, are still needed to understand the complex pathways leading to programmed cell death. This can provide quantitative analysis of the connections between the different cell death types and their role in HI neurodegeneration in the newborn.

Although animal models are critical for studying and understanding the mechanisms of HI injury and for pharmacological testing of potential therapeutics, they are very close but do not completely reflect the pathophysiology observed in a human brain following neonatal HI insult. In the forebrain and cerebellar cortex of the human neonate selective neuronal populations degenerate with no evidence of infarct, with some degenerating cortical neurons staining positive, but some also negative for cleaved caspase-3 [138]. At the same time some degenerating cortical neurons with necrotic morphology appear positive for p53, although such positive cells have not been observed in animal models [138]. Studies of human term brains of infants who suffered perinatal asphyxia and severe HI encephalopathy report enhanced autophagy associated with neuronal death after HI [140, 143]. This overall suggests that classic apoptosis has little contribution to damage occurring in the human brain following neonatal HI and underlines the importance of understanding the mechanisms of the crosstalk between the different types of cell death.

## 9. Gender-Specific Differences in Cell Death following Neonatal Hypoxia-Ischaemia

Most rodent studies looking at the levels of cell death following neonatal HI include both sexes and report combined data. However a lot of clinical and experimental evidence suggests important differences between males and females, with increased loss of male hippocampal volume after chronic postnatal hypoxia and male sex considered a well-established risk factor for poor neurodevelopmental outcome after premature birth [148]. Several studies demonstrate that males are more prone to suffer stroke [149, 150] and have higher incidence of prematurity, intraventricular haemorrhage, and mortality due to prematurity [151, 152]. Clinical studies following very prematurely born infants report male sex as a risk factor for poorer lung function, increased respiratory morbidity, and worse neurological function overall [153]. The mechanisms underlying these gender-related differences are unknown with some evidence suggesting that testosterone exacerbates damage, or that oestrogen/progesterone are protective, or that gender differences in cell death pathways may favour females [152]. Studies of neonatal cerebral ischaemia report involvement of sexually dimorphic pathways of cell death with males predominantly displaying caspase-independent PARP/NO-mediated cell death, resulting in AIF release and translocation, and DNA fragmentation, while females are showing mitochondrial cytochrome C release and subsequent caspase-dependent cell death activation [154, 155]. Thus sex differences are an important parameter that needs to be considered when assessing brain damage following neonatal HI and further studies taking into account these differences need to be conducted for the development of efficient neuroprotective strategies.

## 10. Neonatal Hypoxia-Ischaemia and Plasticity of the Developing Brain

The term plasticity (from the Greek “plastos” meaning moulded) has been introduced by Merriam-Webster as “the capacity to vary in developmental pattern, in phenotype, or in behaviour according to varying environmental conditions.” Brain plasticity includes carefully regulated molecular, cellular, and physiological events promoting the ability of the brain to amend its own organisation and function in response to body changes or environmental alterations. The developing brain is more malleable to external stimuli compared to the adult one, which is generally considered advantageous in respect of recovery of function [156]. Enriching environmental conditions can trigger a positive response in the brain with most beneficial outcomes observed during maturation. In both animals and humans stimulating environment triggers outgrowth of neural projections, resulting in increased dendritic branching and cognitive enhancement [157–159]. Although the developing brain is more plastic and thus would be expected to have better recovery mechanisms following injury, it seems that the immature brain has some of the worst developmental outcomes following significant insult [156]. Injury and seizures trigger excessive stimulation of particular pathways normally involved in shaping the developing brain circuitry, which under these circumstances promote outgrowth of neural projections generating abnormal connections and circuitry and could subsequently lead to epilepsy, motor, and cognitive impairment [21, 156]. For example, glutamate is important for classical neurotransmission, as well as for activity-dependent plasticity during development [160]. While increased amounts of synaptic and extracellular glutamate are observed in most brain regions with glutamate-containing pathways, the toxic effects of glutamate accumulation depend on the type of postsynaptic glutamate receptors.

NMDA receptors are involved in activity-dependent synaptic plasticity, including long-term potentiation (LTP) and refinement of synaptic connection [160, 161]. They require coactivation by glutamate and glycine and are also voltage-dependent, necessitating postsynaptic membrane depolarisation to release the magnesium channel-block thus allowing the NMDA channel to open and calcium to flow into the cell [160]. Therefore the NMDA receptors appear to be particularly important for the pattern of injury in the developing brain, as HI can disrupt the membrane potentials thus overcoming the magnesium block and opening the channels. Functionally the NMDA receptor activity is controlled through changes in the subunit composition [162, 163]. Autoradiographic studies of glutamate binding to NMDA receptor in rat hippocampus demonstrated an excessive increase in receptor density in comparison to adult brain, as well as selective changes in binding to glutamate binding sites and channels [164]. Electrophysiological studies of rat thalamocortical synapses demonstrate that LTP and NMDA-mediated synaptic currents are increased at postnatal day 3 to 7, which is a critical period for somatosensory cortical plasticity [165]. Thus it is quite likely that NMDA receptors are involved in mediating the damage following HI insult to the developing brain and the use of NMDA channel blockers has neuroprotective potential for this type of injury.

AMPA receptors also participate in injury to the developing brain following HI insult. Although AMPA receptors are mostly associated with the trafficking of sodium, immature AMPA channels transport calcium as well. However, in rodents the increasing expression of GluR2 receptor and RNA editing within the first two postnatal weeks generates calcium impermeable AMPA channels [162, 166]. Developmentally the NMDA receptors are the first ones to appear on the newly formed synapse, followed by AMPA receptors associated with increased neuronal activity [160]. AMPA agonists produce greater brain injury in neonatal compared to adult animals, while AMPA antagonists do not demonstrate an immense neuroprotective potential in comparison to NMDA receptor antagonists following HI insult to the developing brain. Both NMDA and AMPA receptors in the immature brain participate in activity-dependent neuronal plasticity and development; however their enhanced function during brain maturation also results in increased vulnerability to excitotoxicity of both neurons and oligodendrocytes. Therefore compared to the adult brain the immature one can survive longer periods of energy depletion due to its lower energy needs; however when this deprivation reaches a certain threshold, excitotoxic pathways are activated and excitotoxic injury escalates [160, 167].

Skoff et al. ultrastructurally studied the neuron-glia interactions in rodents at 1 month following moderate neonatal HI injury, showing that this type of brain insult produces continued degeneration as well as recovery of neuronal and glial elements [63]. The severity of insult directly correlates with the level of degeneration with increased severity being more deteriorating. The contralateral side of the injured animals did not differ from age-matched controls, with lateral cortex containing a mixture of small and large diameter axons, and small and large myelinated fibres, and the striatum appearing normal in most areas with many mature oligodendrocytes and myelinated fibres of normal diameter [63]. Small and thinly myelinated axons, suggesting neuritic growth, were also observed in normal animals, as well as on the contralateral side of HI brains. However, the ipsilateral side demonstrated cortical and striatal bundles of neurites, as well as many immature newly formed and mature synapses, and hardly any astrocytic processes in the bundles [63, 168]. On the ipsilateral side Skoff et al. observed neurons undergoing degeneration even at 1 month after injury and normal axons and axons undergoing degeneration but having normal myelin sheath, suggesting that axonal degeneration is not necessarily secondary to oligodendroglial and myelin degradation [63]. However, they also registered axonal and dendritic growth cones with synapses occasionally attached to them, clearly indicating actively growing neuronal processes and active synapse formation [63]. Thus the ipsilateral side revealed cellular elements of both neural degeneration and recovery often in direct physical proximity, suggesting that some cells remain extremely vulnerable to insult a month after injury while others are spared [63]. The abundance of neurites on the ipsilateral side is a sign for recovery, although it is unclear whether they generate from postmitotic neurons whose development is delayed due to the injury; from neurons with injury-severed axons capable of regenerating new processes; from new neurons projecting their neurites through the lesion site; or from contralateral neurons, whose projections are involved in ipsilateral repair [63]. Overall this data suggests that as all the components and cellular processes required for functional recovery (sprouting of neurites, synapse formation, and myelination) are present in the ipsilateral hemisphere following HI insult in the developing rodent brain, the long-term functional deficits are likely to result from inability of the regrowing axons to innervate their normal targets because of the physical boundaries of the lesion and the abnormal cell types in the injured hemisphere [63].

It has been long established that neural stem cells in the dentate gyrus of the hippocampus and the subventricular zone (SVZ) continue to proliferate during adulthood [169, 170]; however the neurogenesis capacity of the brain decreases with age due to the increasing production of negative regulators [170]. Surprisingly, injury to the brain does not reduce or impair endogenous neurogenesis, but quite the opposite. Neurogenesis is actually preserved or even increased after seizures and stroke in rodent animal models, with evidence suggesting extensive cell proliferation in the SVZ following HI [170]. Several studies demonstrate that 1–3 weeks after moderate HI the SVZ expands in size, with an increased number of 5-Bromo-2′-deoxyuridine (BrdU) positive cells, suggesting higher levels of proliferation. BrdU positive cells are also registered in cortex and striatum, probably due to either migration of proliferating cells from the SVZ or increased capacity of the local progenitors to proliferate in response to the injury-triggered environmental changes [170]. However, despite the endogenous neurogenesis capability of the brain, a HI insult during or around the time of birth would still cause injury due to excessive cell loss or as a result of impairment of growth and differentiation factors production [170]. Some compounds pharmacologically reducing neuronal cell death and inflammation have a longer therapeutic window probably because they promote neuronal migration, neurogenesis, and oligodendrogenesis [171, 172]. Several groups suggest the use of stem cell treatment as an opportunity to increase the capacity of the neonatal brain to regenerate [173], mainly by the use of mesenchymal stem cells (MSCs) [170, 174, 175]. MSC transplantation following neonatal HI has proven neuroprotective, although the precise mechanism behind that effect is not clear. MSCs are able to migrate to site of injury, differentiate into specific lineages, and possess anti-inflammatory properties, thus aiding brain tissue repair through possible replacement of damaged neurons and oligodendrocytes, and modulation of the host inflammatory response. Another possibility is that the MSCs do not integrate in the host network but stimulate the proliferation and differentiation of endogenous precursors [170]. Thus MSC therapy has a high potential for treatment of neonatal HI brain damage through stimulation of the endogenous neuroregeneration and plasticity.

Apoptosis and caspase activation play a very important role in the developing brain for elimination of redundant and damaged neurons and sculpting the tissue. HI injury interrupts the apoptosis signalling due to mitochondrial structural and functional failure, thus resulting in the occurrence of the apoptosis-necrosis continuum [119]. Around the time of birth cortex and basal ganglia undergo dynamic development, associated with shaping of central motor pathways, involving establishment of new corticothalamic connections, as well as elimination of old ones [21]. A HI event around this time interrupts these processes and depending on its severity can affect normal developmental plasticity through altering neurotransmission, changes in cellular signalling, neural connectivity and function, and wrong targeted innervation. Studies looking at traumatic injury in the developing brain [156], which in a way resembles HI insult, have pointed out that the dogma “younger is better” may be incorrect and that “good” plasticity under traumatic conditions can be transformed into “bad” plasticity. Understanding the mechanisms behind this transformation would allow more effective approach towards treatment following HI injury in the developing brain and possible prevention of the subsequent neurodisabilities.

## Figures and Tables

**Table 1:** Cell death phenotypes.

	Cytoplasm	Cell membrane	Nucleus
Apoptosis	(i) Condensation (ii) Shrinkage (iii) Undamaged mitochondria, but might also appear swollen	Undamaged	(i) Large chromatin clusters (ii) Undamaged nuclear membrane

Necrosis	Totally disrupted organelles	(i) Rupture (ii) Swelling	(i) Undamaged nuclear membrane (ii) Widely scattered very small chromatin clusters

Apoptosis-necrosis continuum	(i) Varying degrees of condensation (ii) Rarefaction with varying preservation of organelles (iii) Undamaged mitochondria (iv) Occasional autophagocytic inclusions	Undamaged	(i) Incomplete packaging of nuclear chromatin into small and more numerous clusters (ii) Various degrees of membrane preservation
